# Labeled as “drug-seeking”: nurses use harm reduction philosophy to reflect on mending mutual distrust between healthcare workers and people who use drugs

**DOI:** 10.3389/fpubh.2023.1277562

**Published:** 2023-10-16

**Authors:** Sarah Febres-Cordero, Rebecca O. Shasanmi-Ellis, Athena D. F. Sherman

**Affiliations:** Nell Hodgson Woodruff School of Nursing, Emory University, Atlanta, GA, United States

**Keywords:** nursing, harm reduction, philosophy, drug-seeking, stigma, discrimination, healthcare barrier, healthcare providers

## Abstract

**Introduction:**

Over 50 years of approaching drug use from the “War on Drugs” has led to ignoring the systemic structural and social determinants of health, enforced drug use stigma, and damaging stereotypes of people who use drugs or are labeled as “drug-seeking,” and sorely failed to support those needing assistance.

**On philosophy of harm reduction and power:**

People who use drugs are often disenfranchised and pathologized by being labeled as “a drug addict,” which then serves as a rationalization for mistreatment by healthcare providers. This is in opposition to a harm-reduction approach. Harm reduction philosophy is an epistemic valuation necessary for drug use stigma and our moral obligation to reduce harm from interlocking systems of power that perpetuate harm.

**On drug-seeking, mistrust, and human rights:**

We have encountered many clients who use drugs that report harmful interactions with healthcare providers. Harm reduction is an issue of health equity, social justice, and fundamental human rights. This paper presents three vignettes, the author’s experiences of being labeled as—and advocating for family members labeled as “drug-seeking.”

**Discussion:**

To better serve as healthcare providers, workers must be equipped to work with people who use drugs and reinforce the social justice commitment against medical stigma, neglect, racism, and inadequate pain coverage and withdrawal treatment. Nurses and our epistemic lens can meet the challenge of complex intersectional issues affecting our use of power to develop more just and equitable health systems and advance our rebuilding of a trusting relationship with the people we serve.

## 1. Introduction

For better or worse, drug use is part of our world (1). For decades in the United States (US), we have identified people who use drugs as an enemy to be destroyed (i.e., the “War on Drugs”) or an entity to be ignored (e.g., “Just Say No”) (2–5). This rhetoric has created a widespread multi-level (individual, interpersonal, and structural) stigma aimed at people who use or are addicted to drugs in the US, particularly among Black people, Indigenous people, and other people of color (BIPOC) and Latinae communities (6–10). Stigma contributes to the avoidance of help and treatment-seeking, risks of social exclusion, loss of social support or employment, and potential incarceration complicate health promotion and premature death among those who use drugs (11).

Although many say the “War on Drugs” is ending, this stance has led to decades of structural racism, mass incarceration, generational poverty, and loss of life—as people conceal drug use and addiction related to stigma and fear of punitive policies (10–12). In the US, the ability to address recreational and excessive drug use has been pushed back onto the individual, leading to increased shame and self-deprecating behaviors (13, 14). Such approaches, which ignore the systemic structural and interpersonal determinants of health, enforce drug use stigma and damaging stereotypes of people who use drugs and sorely fail to support those needing assistance (15, 16). In response to complex issues in healthcare such as this, innovative, culturally aware approaches are necessary to repair the delivery of nursing care and, ultimately, improve health outcomes for disenfranchised communities (e.g., people who use or are addicted to drugs). To do so, Canty et al. (17) have called for the inclusion of diverse voices into the nursing philosophy discourse. Thus, our diverse team of nurses (i.e., a Hispanic nurse, a Black nurse, and a queer pansexual gender fluid nurse) has gathered to provide our insight on harm reduction and power in the care of people who use drugs and those perceived as drug-seeking by sharing our experiences of being labeled as drug-seeking with healthcare providers.

## 2. On philosophy of harm reduction and power

Healthcare workers are trained to treat and cure people. People who use or are addicted to drugs seek recovery when ready. In the meantime, they also get sick and injured like everyone else. However, they are not given the same respect, dignity, and compassion that people who do not use drugs receive (18). They are often disenfranchised and pathologized by being labeled as “a drug addict,” which then serves as a rationalization for mistreatment by healthcare providers and staff (19). This opposes a harm-reduction approach, which is “a set of practical strategies and ideas aimed at reducing the negative consequences associated with drug use and is a movement for social justice built on a belief in, and respect for, the rights of people who use drugs” (1). Philosophers have discussed harm reduction as an epistemic valuation necessary for our discourse and moral obligation to reduce harm from interlocking systems of power that perpetuate harm (20). The paradigm shift to harm reduction in nursing borrows from Foucault’s emphasis on the nurses’ role and agency in using their power to promote and protect human dignity and human rights (21, 22).

## 3. On drug-seeking, mistrust, and human rights

Once someone becomes physically or emotionally dependent on drugs, they may require medicines to be free from withdrawal symptoms (23). This dependence, or need for medications, can be all-consuming, leading to injecting drugs in public spaces and practicing risky drug use behaviors (e.g., using unhygienic supplies) (24, 25). For some, this need brings them to emergency departments (ED) and healthcare providers, seeking relief from the pain and illness associated with withdrawal. Thus, nurses are often the first point of contact between people who use drugs and the healthcare system.

Over the years, the relationship between healthcare workers and people who use drugs has become increasingly strained. Healthcare workers may look out for drug-seeking behaviors, and when they encounter people who use and are addicted to drugs, they may assume drug-seeking is the reason they are seeking care (18). Additionally, once they recognize stereotypically stigmatized symbols of drug use (injection marks, abscesses), they often fail to see the humanity of the client, respond with discrimination or victimization, and may reinforce drug use stigma by labeling the client as a “drug user” or “drug seeking” (26). The term drug-seeking alone carries stigma and judgment, as seeking “drugs” implies a want rather than a need.

As harm reduction nurses, nurse scientists, and nurse educators, we have encountered many clients who use drugs that report harmful interactions with healthcare providers. The stories are different, but the one thing they all had in common was being grossly mistreated by nurses, doctors, and other healthcare providers. Moreover, those in recovery from harmful drug use often report needing to hide their past drug use due to the extreme stigma experienced by healthcare workers. Stories of neglect and discrimination toward people who use drugs are shared in qualitative research (18, 26, 27) and perspectives by healthcare providers (28) who see that both external (18) and internalized (28) stigma act as barriers to healthcare for people who use drugs (26, 29, 30).

These stories of mistrust may act as motivation to train healthcare providers to shift to a philosophy of harm reduction and become harm reduction practitioners, treating all with dignity, respect, and compassion (20, 31–33). Nurses are the most trusted profession, and doctors come in as a close second, except among people who use drugs (34). This mistrust has come from years of abuse and neglect (18, 29, 30, 33). The irony of this mistreatment of people who use drugs is that as many as 11–20% of the healthcare workforce is thought to have a substance use disorder (35, 36).

### 3.1. Vignettes

Some may say it is extreme to say that healthcare providers, especially nurses, abuse people who use drugs. Unfortunately, our team has experienced stigma related to drug use and suspected drug-seeking behavior in the form of mistreatment and neglect from nurses, midwives, and doctors. Below we detail three vignettes as evidence of the harmful nature of perpetuated drug use stigma in healthcare.

#### 3.1.1. Vignette, SFC: trauma and pain in the ED

In the winter of 2021, I was in an accident. I sustained a displaced tibial plateau fracture while out with my family. I somehow got into a vehicle and was dropped off at the ED (COVID precautions kept anyone from joining me). I had no one to advocate for me in the ED. It was just me. My partner retrieved a wheelchair from inside and left me with the triage nurses. I was in tremendous pain, although from outward appearances, I looked fine. There was no swelling at the site, and the fracture was not visible. I was brought to triage, and I was crying and upset. I had experienced a trauma; I was in shock. In triage, the nurses asked me to consent to an X-ray. I asked if I would have to get onto a table for an X-ray. They answered in the affirmative. It was then that I said, I am a nurse. I know that to get onto that table, to have that procedure (the X-ray), I will need pain medication first. This is the standard teaching in the profession. I told them I could not get on the table without medication.

The nurses disagreed. They insisted that I try. I cried and begged for medication before the X-ray. I was denied. A nurse came to take me to the X-ray machine. I could not do it. The pain was unbearable. I have never screamed the way I did that day. I felt like a wounded animal, acting instinctually to protect my leg. I screamed, and I cried. I later wondered who could hear my screams throughout the hospital; they were terrible. The nurse laughed at me while struggling to get on the table and told me I was an amazing actress. She looked at me with disgust.

I could not do it. I was taken back to the empty waiting room and told it might be a while before they could take me to a bed and bring a mobile X-ray machine. I do not know how long I sat there, I was exhausted, saddened, and all I wanted was someone to help me. By being my advocate for pain relief, with no visible signs of injury, it seemed that I had been labeled a drug seeker.

Eventually, I was taken to a bed and received an X-ray and diagnosis. I had a new nurse. He apologized for the delay in the X-ray and pain medication. I called one of my colleagues, and they stayed with me, on the phone, for the rest of my time in the ED. They advocated for proper pain management and compassionate care. At one point, the nurse who abused me walked by my door; I saw her look into my room. Our eyes met for a moment, and then she walked away. I never received a formal apology from her or the hospital.

#### 3.1.2. Vignette, ADFS: postpartum injury and denial of pain treatment

I had recently returned home after an unplanned c-section and prolonged hospital stay with my firstborn child. Seven days postpartum, I fell down a flight of stairs holding my newborn. I clutched my child to my chest to shield them from the fall and took the brunt with my back as I slid down the 8–10 stairs before stopping at the bottom floor. In a rush of terror and adrenaline, I screamed for my partner, who ran to our aid.

I could not stand, and my partner helped me sit on the floor while they looked over our newborn for injury. We immediately called the pediatrician for guidance and were assured that since the newborn’s crying subsided within moments of being calmly held, they could be monitored at home and brought in for their regular check-up at the end of the following week.

The adrenaline began to subside once that emergency was resolved, and the pain set in. I had increased vaginal bleeding, extreme 10/10 pain at the surgical site with any attempted movement, and 7/10 throbbing pain while at rest. The pain made my head spin and nausea unbearable. We called the obstetrician, and they told me to come in immediately for an ultrasound to confirm if the surgical site had been compromised.

I cried in pain as I sat in the operatory, waiting for my ultrasound to be read. Finally, the clinician arrived, confirmed the site had not ruptured, and insisted that I could not be in the amount of pain I was reporting. I asked what I could safely take for pain management while breastfeeding. The provider laughed and said they would not be prescribing any medications and to handle my symptoms using Tylenol. They insisted that my pain was falsified. Finally, I broke down and asked to speak to another provider.

Shortly after, the lead clinician of the practice came in to assess me. After only minutes, they confirmed that my pain was real and valid and called a 2-week medication regimen to manage the pain and nausea and set a 1-week follow-up to adjust the regimen as needed.

#### 3.1.3. Vignette, RSE: chronic pain among older adults, bullying, and denial of respectful care

My mother, at age 72, was diagnosed with Shingles. This resulted in a 3-day hospital stay and her being put on home oxygen for 1.5 years because the location of her Shingles exacerbated her existing lung condition. Working with her pulmonologist, we worked hard for her to regain better lung function, but during this time, she was also diagnosed with Postherpetic Neuralgia—a chronic pain condition caused by Shingles. The pain specialists told us she had the worst and most prolonged case they had seen. Flare-ups at her Shingles site sent her into pain so bad she could not breathe.

One of the reasons she and my late father, immigrants to the US, bought their home was because they were aging, and the house was across the street from a community hospital. One night a flare-up sent her to the ED. Being received at the hospital at 2:00 AM, she was made to wait 4 hours in a room alone with elevated blood pressure, heart rate, and decreased oxygenation, with no pain relief. The only attention given to her was a nurse coming in to assess her pain score, who repeatedly and forcefully said, “Your pain score is a 2 [out of 10], right.” Confused and in pain, my mom kept crying out—telling the nurse, “I need a doctor. The pain is so bad,” but she was bullied into saying yes to a 2/10 pain score.

Even though this was a hospital she had been a patient of for 15 years, their chart review, or even her medication reconciliation sheet she carried everywhere, would show them she was not on any narcotics for her chronic pain. This was ignored because she was labeled as “drug-seeking.” She was bullied, and documentation stated that no medical intervention was needed. Nothing would be done for her care until I arrived at 6:00 AM and advocated for respectful care.

## 4. Discussion

As seen in our past experiences, the stigma of drug-seeking has permeated beyond those who seek drugs. Any stigmatized symbol of drug use (both present and past) and knowledge of pain medications may be interpreted as drug-seeking. People who are knowledgeable about drugs and their use for pain, or a preference for a drug or pain treatment, may be dismissed as drug-seeking (37). Additionally, subjective cues such as race and ethnicity have been found to affect prescribing opioids and potent painkillers, as people of color are more often labeled as drug-seeking (38). This and other factors, including racism, have led to the undertreatment of pain among minorities (39–45). This was evidenced in the above encounters. Mistreatment related to drug use stigma discounts patient autonomy, human dignity, and the right to advocate for themselves—thus, often relying on a third party to confirm the person’s need for pain relief (20, 22). Removing autonomy and individual advocacy in patient care is neglectful and constitutes an abuse of authority in healthcare—perpetuating the paternalistic structures in the healthcare system (46, 47).

### 4.1. Toward a harm reduction nursing philosophy

To better serve as healthcare providers, we need to equip workers to work with people who use drugs, especially in the ED, where they often have their complex health needs addressed (48). Risjord (21, p. 36) presents from an epistemic standpoint, nurses, having a “political commitment to justice [while valuing their] role, [can] question the dominant account of society” regarding the stigma of drug use. Harm reduction philosophy allows nurses to integrate their commitment with empirical knowledge (21). Harm reduction benefits nurses’ work by offering evidence-based public health strategies that align with nurses’ ethical principles of autonomy (the right to self-determination), justice (treating all with dignity, respect, and humanity), and non-maleficence (avoiding or minimizing harm), and makes it possible for people who use drugs to seek non-judgmental care (rather than avoiding healthcare providers), considering the reality of peoples’ lives and experiences (20). This makes a way forward to manage pain, healthcare, and well-being for people who use drugs, are in recovery, or are suspected of using drugs.

### 4.2. Approaches to rebuilding trust with disenfranchised communities

Mistreatment by medical teams has deleterious effects, including denial of care, reduced help-seeking, and disengaging treatment against medical advice when faced with stigma, neglect, racism, and inadequate pain and withdrawal treatment. The well-being of our patients necessitates us to support people who require healthcare, pain relief, and support from withdrawal to ensure they can receive the treatment they need. Varied approaches to rebuilding trust between disenfranchised communities and healthcare systems exist, including forming trusting relationships with public health and social service agencies to improve population health, having a clear vision for providing services that address social determinants of health, investing in communities by providing jobs and resources for the community and taking the time to understand the communities you wish to serve (49, 50). Additionally, people who use drugs must have a seat at the table when tailoring services for them. The “nothing about us without us movement” among people who use drugs insists that they be included in decision-making that impacts their care and is essential in regaining trust (51). Lastly, healthcare providers should engage in shared decision-making where people who use drugs can advocate for themselves to receive the care they require and want, supported by healthcare providers (52, 53).

### 4.3. Global human rights policy and reform

Systemic change is needed to increase access to care and expand resources while creating policies favorable to harm reduction (54). Globally, there has been an expressed need to expand harm reduction services to manage drug-related harm and inform healthcare interventions and policies (55). Policies and laws still act as barriers to providing harm reduction services for those at risk of drug-related harm. A 2023 systematic review of global harm reduction services for people who use drugs (syringe exchange, opioid agonist treatment, supervised consumption facilities, naloxone distribution, and drug-checking services) found that among the countries with evidence of drug injecting, many were lacking one or more harm reduction services for this population (55). Globally, structural barriers to implementing and accessing such services included barriers to funding, fear of arrest for drug use or possession of injecting equipment, stigma and discrimination, and lack of trust in government (55).

Additionally, as highlighted by African country contexts, recommendations for providing healthcare and harm reduction services for people who use drugs must consider the need for community-based approaches to accessing care (56). These include drop-in centers, mobile outreach, clinics, peer-led outreach, and community-led services while including people who use drugs in the development of policy and programs (56). Kenya’s context and its advances in making a systemic change toward a harm reduction approach to people who use drugs have led it to be the largest and most widespread service delivery program in Africa, addressing the comprehensive health and wellness needs of people who use drugs as part of that country’s national strategic plan. Kenya’s programs have seen between 70 and 98% retention rates (56).

Though there have been global attempts at strategizing to improve the healthcare workforce specifically, concrete interventions are still lacking in countries like Australia (57). In Portugal, changing national policies to focus on drug use as an issue of health rather than a criminal issue by decriminalizing personal drug use and implementing harm reduction strategies has led to a greater understanding of why people use drugs. The work in Portugal has shown that 90% of people who use drugs do not have a substance use disorder (58), and people use drugs in functional, non-dependent, religious, healthy, socially integrated, and non-problematic ways (59). This global humanitarian view of drug use behaviors could aid healthcare providers in the US who quickly pathologize and stigmatize drug use to take a more human-centered, holistic view of people who use drugs. Additionally, the work being done in Portugal has led to the acknowledgment that individuals who use drugs should have the autonomy to consent to any mandates, clinical evaluations, diagnosis, and treatment as part of their human rights (59).

### 4.4. Harm reduction for health equity

As highlighted in this paper, we cannot separate the “messy empirical realm of real-world harms” (20). Decades of advocacy have gotten us to move from the punitive lens of the war on drugs, abstinence approaches, and “just say no,” which have failed us, toward harm reduction philosophy with the opioid epidemic. We have a shared humanity, whether we use drugs or not. Those who get sick and sustain injuries require healthcare and deserve healthcare, regardless of drug use.

Harm reduction is an issue of health equity, social justice, and fundamental human rights. Nurses and our epistemic lens can meet the challenge of complex intersectional issues affecting our use of power to develop more just and equitable health systems and advance our relationship with the people we serve. We urge all nurses and healthcare providers to consider a harm reduction approach to working with people who use drugs. Harm reduction is a human rights approach to caring for people who use drugs. Nurses have an ethical duty to treat people with dignity, respect, and compassion, regardless of their drug use. By meeting people where they are, providing non-judgmental care, and providing non-coercive provision of services, we can work toward rebuilding a trusting relationship with people who use drugs.

## Data availability statement

The original contributions presented in the study are included in the article/supplementary material, further inquiries can be directed to the corresponding author.

## Author contributions

SF-C: Conceptualization, Writing – original draft, Writing – review & editing. RS-E: Conceptualization, Writing – original draft, Writing – review & editing. ADFS: Conceptualization, Writing – original draft, Writing – review & editing.
